# Tandem trimer pyrrole–imidazole polyamide probes targeting 18 base pairs in human telomere sequences†
†Electronic supplementary information (ESI) available: Materials and methods, sequences of ODN-1 to 5, mass spectra and analytical HPLC profiles of the compounds **TH59**, **TT59**, **TAMRA TH59** and **TAMRA TT59**, the UV-visible absorption and fluorescence spectra of probes **TAMRA TH59** and **TAMRA TT59**, SPR sensorgrams of **TH59** and **TT59**, additional images of telomeres. See DOI: 10.1039/c4sc03755c



**DOI:** 10.1039/c4sc03755c

**Published:** 2015-01-20

**Authors:** Yusuke Kawamoto, Asuka Sasaki, Kaori Hashiya, Satoru Ide, Toshikazu Bando, Kazuhiro Maeshima, Hiroshi Sugiyama

**Affiliations:** a Department of Chemistry , Graduate School of Science , Kyoto University , Kyoto 606-8502 , Sakyo , Japan . Email: hs@kuchem.kyoto-u.ac.jp ; Email: bando@kuchem.kyoto-u.ac.jp; b Biological Macromolecules Laboratory , Structural Biology Center , National Institute of Genetics, and Department of Genetics , School of Life Science , Graduate University for Advanced Studies (Sokendai) , Mishima , Shizuoka 411-8540 , Japan . Email: kmaeshim@nig.ac.jp; c Institute for Integrated Cell-Material Science (WPI-iCeMS) , Kyoto University , Kyoto 606-8501 , Sakyo , Japan

## Abstract

The binding of molecules to specific DNA sequences is important for imaging genome DNA and for studying gene expression. Increasing the number of base pairs targeted by these molecules would provide greater specificity. *N*-Methylpyrrole–*N*-methylimidazole (Py–Im) polyamides are one type of such molecules and can bind to the minor groove of DNA in a sequence-specific manner without causing denaturation of DNA. Our recent work has demonstrated that tandem hairpin Py–Im polyamides conjugated with a fluorescent dye can be synthesized easily and can serve as new probes for studying human telomeres under mild conditions. Herein, to improve their selectivities to telomeres by targeting longer sequences, we designed and synthesized a fluorescent tandem trimer Py–Im polyamide probe, comprising three hairpins and two connecting regions (hinges). The new motif bound to 18 bp dsDNA in human telomeric repeats (TTAGGG)_*n*_, the longest sequence for specific binding reported for Py–Im polyamides. We compared the binding affinities and the abilities to discriminate mismatch, the UV-visible absorption and fluorescence spectra, and telomere staining in human cells between the tandem trimer and a previously developed tandem hairpin. We found that the tandem trimer Py–Im polyamide probe has higher ability to recognize telomeric repeats and stains telomeres in chemically fixed cells with lower background signal.

## Abstract 2

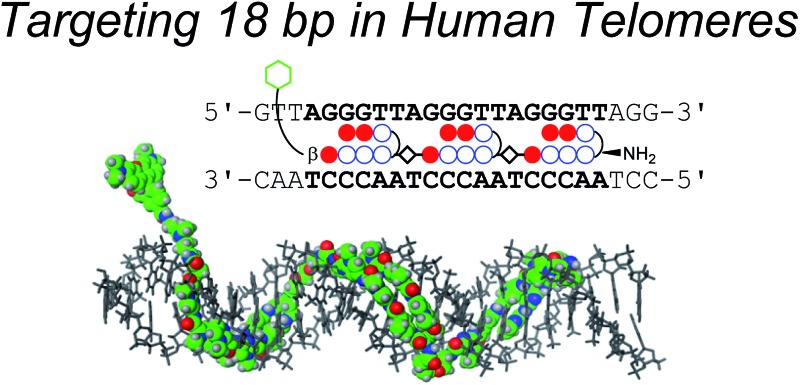
The novel tandem trimer pyrrole-imidazole polyamide probe targeting 18 bp in telomeric repeats visualized telomeres in human cells selectively.

## Introduction

Telomeres are localized at the ends of chromosomes and play important roles in the stability and replication of chromosomes.^1^ Human telomere DNA, whose sequences are the tandem repeats of 5′-TTAGGG-3′, has a duplex region and a 3′ overhang, which tends to form G-quadruplexes or t-loops.^2^ In normal mammalian cells, the number of telomere repeats decreases with cell division, and this decrease is related to the aging process and cancer.^3,4^ Therefore, the telomere length is one important biomarker used in various diagnoses.^2–5^ Fluorescent probes targeting telomeres have been developed to visualize and measure the telomere length, which is regulated by the shelterin complex.^6^

Our group has focused on *N*-methylpyrrole (Py)–*N*-methylimidazole (Im) (Py–Im) polyamides, as reported by Dervan and co-workers. Py–Im polyamides bind strongly and sequence-specifically to the minor groove of dsDNA by recognizing Watson–Crick base pairs without causing DNA denaturation.^7^ The antiparallel Im–Py pair recognizes a G·C base pair, whereas a Py/Py pair recognizes an A·T or T·A base pair.^7a,b^ C-terminal β-alanine and the γ-turn moiety comprising γ-aminobutyric acid or 2,4-diaminobutyric acid (Dab) also recognize A·T or T·A base pairs, and (*R*)-Dab affords a higher binding affinity comparable to that of γ-aminobutyric acid.^8^

Py–Im polyamides can be synthesized by machine-assisted Fmoc solid-phase peptide synthesis (SPPS).^9^ Various structures of Py–Im polyamides, such as linear,^10^ hairpin,^11^ and cyclic,^12^ have been synthesized to provide efficient binding. In particular, hairpin Py–Im polyamides are versatile and can be used as sequence-specific functional conjugates.^13^ To gain specificity, Py–Im polyamides targeting longer sequences (≥10 bp) have been developed.^10,14–17^ DNA ligands recognizing ≥16 bp are required to target a single site in the human genome, which comprises about 3 billion base pairs. Dervan and co-workers reported a linear Py–Im polyamide that formed a homodimer with two molecules and then bound to 16 bp sequences in the regulatory region of the HIV-1 genome.^10*b*^ Our laboratory has reported a cysteine-derived Py–Im polyamide dimer that binds to 16 bp with one molecule.^14*c*^

Tandem hairpin Py–Im polyamides targeting ≥10 bp, which comprise two hairpin moieties and a hinge segment between two hairpins, have been developed.^15,16^ For instance, Maeshima, Janssen, and Laemmli described tandem hairpin Py–Im polyamide probes that could bind to 12 bp sequences in the duplex region of the human telomere repetitive sequence TTAGGG and could stain telomeres in chemically fixed cells.^16^ Solid-phase synthesis requires many steps, which leads to a lower yield, and there are fewer reports on the targeting longer sequences with Py–Im polyamides than those described for the binding of conventional polyamides to shorter sequences.

**Fig. 1:**
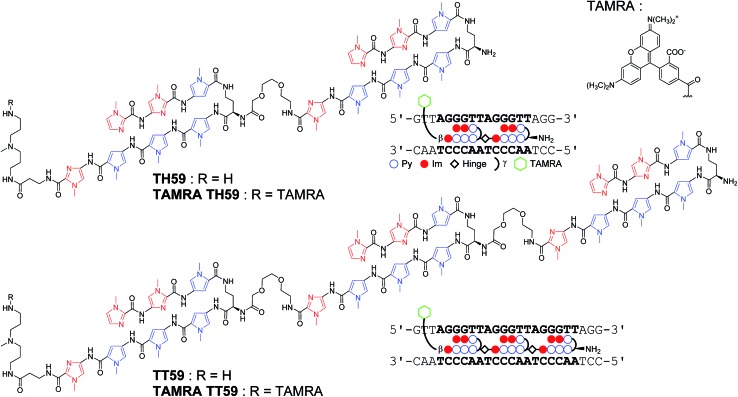
Chemical structures of Py–Im polyamides **TH59**, **TT59**, **TAMRA TH59** and **TAMRA TT59** targeting the human telomere sequence and their ball-and-stick representations.

Recently, our group proposed a new method for the synthesis of tandem hairpin Py–Im polyamide **TH59** (Fig. 1) using a new building block. The block comprises the sequence from Dab to the N-terminus of a hairpin moiety, and is used as a unit of SPPS to decrease the procedure on solid support. Using this methodology, we synthesized fluorescent telomere probes^17a,b^ and alkylation agents.^17c,d^ Human telomeres bound by the probes could be costained with anti-TRF1 antibody under mild conditions without breaking the structure of the telomeres, showing that the telomere length was related to the abundance of the shelterin complex.^17*a*^ This point is an advantage over the PNA FISH method, which requires denaturation under harsh conditions.^6*b*^ More recently, the fluorescent group and the hinge region were optimized to target telomeres in cells more selectively.^17*b*^

Herein, as one of the optimizations of the Py–Im polyamide probes, we describe our synthesis of the new type of Py–Im polyamides, defined as tandem trimers, whose components are three hairpin subunits and two hinges. Our aim was to increase their selectivities to telomeres. After the optimization of SPPS, we synthesized the new tandem trimer Py–Im polyamide **TT59**, which targeted 18 bp in the human telomere repetitive sequence TTAGGG. To our knowledge, 18 bp is the longest specific binding sequence for Py–Im polyamides. Surface plasmon resonance (SPR) analysis was used to assess the binding affinity and specificity of new Py–Im polyamide **TT59** toward telomeric repeat sequences. After conjugation of **TH59** and **TT59** with fluorescent 5-carboxytetramethylrhodamine (TAMRA), we obtained fluorescent telomere probes **TAMRA TH59** and **TAMRA TT59**, respectively (Fig. 1). We found that the new tandem trimer probe **TAMRA TT59** stained telomeres in chemically fixed cells and produced a lower background because of the reduction in nonspecific binding compared with the conventional probe **TAMRA TH59**. These results suggest that the new longer Py–Im polyamides recognize the target sequences in cells with greater specificity.

## Results and discussion

### Synthesis of Py–Im Polyamides

**Scheme 1:**
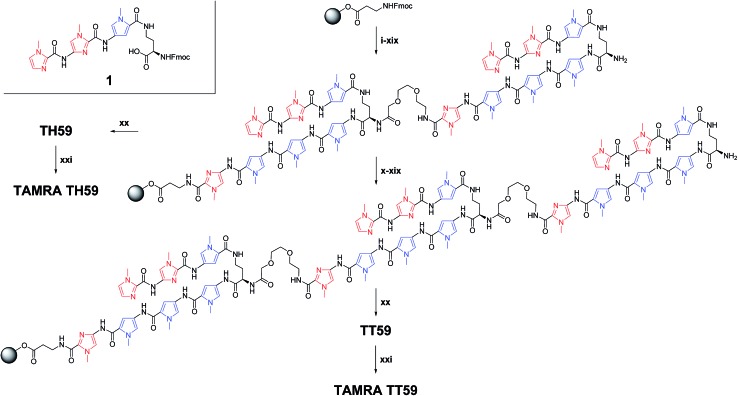
Solid-phase synthesis of Py–Im polyamides **TH59**, **TT59**, **TAMRA TH59** and **TAMRA TT59** and structure of building block **1**. Reagents and conditions: (i) 20% piperidine, NMP; (ii) Fmoc–PyIm–CO_2_H, HCTU, DIEA, NMP; (iii) 20% piperidine, NMP; (iv) Fmoc–Py–CO_2_H, HCTU, DIEA, NMP; (v) 20% piperidine, NMP; (vi) Fmoc–Py–CO_2_H, HCTU, DIEA, NMP; (vii) 20% piperidine, NMP; (viii) **1**, HCTU, DIEA, NMP; (ix) 20% piperidine, NMP; (x) Fmoc–mini-PEG–OH, HCTU, DIEA, NMP; (xi) 20% piperidine, NMP; (xii) Fmoc–PyIm–CO_2_H, HCTU, DIEA, NMP; (xiii) 20% piperidine, NMP; (xiv) Fmoc–Py–CO_2_H, HCTU, DIEA, NMP; (xv) 20% piperidine, NMP; (xvi) Fmoc–Py–CO_2_H, HCTU, DIEA, NMP; (xvii) 20% piperidine, NMP; (xviii) **1**, HCTU, DIEA, NMP; (xix) 20% piperidine, NMP; (xx) 3,3′-diamino-*N*-methyldipropylamine, 55 °C; (xxi) 5-carboxytetramethylrhodamine, succinimidyl ester, DIEA, DMF.

Based on the previous methodology using the synthesized building block **1** corresponding to (*R*)-2,4-diaminobutyric acid turn and three-ring cycle polyamide at the side of N-terminal as one of the units,^17*a*^ synthesis of fluorescent Py–Im polyamide probes was performed after optimization (Scheme 1). These syntheses have suffered from low yield, presumably due to the contamination of moisture. Therefore, the solid-phase peptide synthesizer was placed in a vinyl box with dehumidifiers. Machine-assisted Fmoc SPPS began from Fmoc–β-Ala–Wang resin. Each Fmoc group was deprotected 20% piperidine/1-methyl-2-pyrrolidone (NMP) and then the deprotected amino group reacted with the next Fmoc unit (Fmoc–Py–OH, Fmoc–PyIm–OH, Fmoc–mini-PEG–OH or **1**) activated with *N*,*N*-diisopropylethylamine (DIEA) and HCTU in NMP. Procedures (i)–(xix) were performed for the synthesis of tandem dimer of hairpins on solid support. Though conventional **TH59** could be obtained through the cleavage of the dimer from resin with 3,3′-diamino-*N*-methyldipropylamine, one repeat of procedures (x)–(xix) was required for new tandem trimer **TT59**. As a result, the synthesis of tandem trimer Py–Im polyamide **TT59** succeeded for the first time with an 8.5% yield for SPPS, and the yield of the previous tandem hairpin Py–Im polyamide **TH59** increased from 6.9% (ref. 17*a*) to 29%. The obtained Py–Im polyamides **TH59** and **TT59** were conjugated with 5-carboxytetramethylrhodamine succinimidyl ester in *N*,*N*-dimethylformamide (DMF) and DIEA to afford the fluorescent Py–Im polyamide probes **TAMRA TH59** and **TAMRA TT59**, respectively. Protection of the α-amino group of Dab was not required because of steric hindrance. The ESI-TOF MS spectra and analytical HPLC profiles of **TH59**, **TT59**, **TAMRA TH59** and **TAMRA TT59** are shown in Fig. S1 and S2,† respectively.

### Optical characteristics of the fluorescent probes

The UV-visible absorption spectra of probes **TAMRA TH59** and **TAMRA TT59** are shown in Fig. S3A.† The absorption maximum at ∼555 nm was due to the fluorescent group TAMRA. The absorption maximum at ∼310 nm was derived from the *N*-methylpyrrole and *N*-methylimidazole moieties.

Fig. S3B† shows the fluorescence spectra of probes **TAMRA TH59** and **TAMRA TT59** in the absence and presence of 1.0 or 2.0 equivalent oligonucleotide (ODN)-1/2, whose sequences are shown in Table S1.† Addition of ODN-1/2 to both probes provided little change in the fluorescence intensity and quench of TAMRA was not observed in the absence of ODN-1/2. In contrast, Dervan, Gray and co-workers showed that TAMRA connected to *N*-methylpyrrole in the polyamide backbone was quenched because of the electron transfer from Py to TAMRA in the absence of dsDNA.^18^ It is presumed that the distance between TAMRA attaching to C-terminal through 3,3′-diamino-*N*-methyldipropylamine linker and polyamide was longer and therefore the electron transfer triggering quench became difficult to occur.

### Binding affinities to match sequence and discrimination of mismatch sequences

Binding affinity and discrimination of **TH59** and **TT59** were evaluated with SPR experiments.^17b,19^ Three kinds of 5′-biotinylated hairpin DNAs ODN-3, 4 and 5 containing the binding site of **TT59** were prepared by the immobilization to sensor chips through a biotin–avidin system. The sequences are shown in Table 1 and S1.† ODN-3, 4 and 5 had no mismatch, 1 bp mismatch at the C-terminal of **TT59** and 1 bp mismatch at the center of **TT59**, respectively. Tandem hairpin **TH59** and trimer **TT59** were passed to DNAs on sensor chips and resulting sensorgrams (shown in Fig. S4 and 5†) and values (summarized in Table 1) were obtained. Comparing the values of **TT59** in the matching sequence with those of conventional tandem hairpin **TH59**, the association of **TT59** was slower and thus its binding affinity was weaker. One considerable reason was that bigger structure of tandem trimer **TT59** decreased its own accessibility to the matching sequence. However, since the *K*_D_ value of **TT59** and ODN-3 was 3.6 × 10^–9^ M, the tandem trimer had high binding affinity comparable with other Py–Im polyamides.^7^ To evaluate the discriminations, specificities were calculated by dividing the *K*_D_ value of the mismatch DNA (ODN-4 or 5) by that of the match DNA (ODN-3). The specificity value of tandem trimer **TT59** was bigger than that of **TH59**, suggesting that extending the targeted base pairs could increase the sequence specificities.

**Table 1:** Binding affinities of polyamides **TH59** and **TT59** against match sequence (ODN-3) or 1 bp mismatch sequence (ODN-4 or 5)^*a*^

	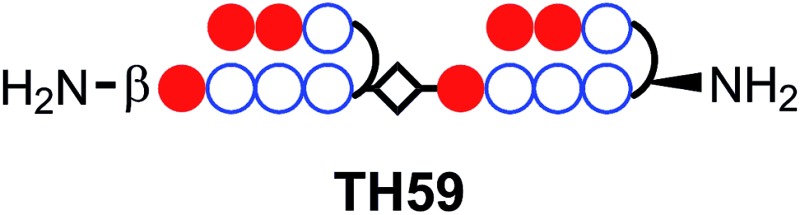					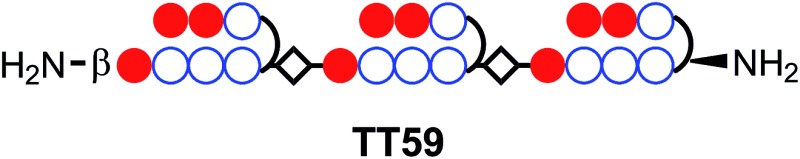				
	*k*_a_ (M^–1^ s^–1^)	*k*_d_ (s^–1^)	*K*_D_ (M)	*χ* ^2^	Specificity	*k*_a_ (M^–1^ s^–1^)	*k*_d_ (s^–1^)	*K*_D_ (M)	*χ* ^2^	Specificity
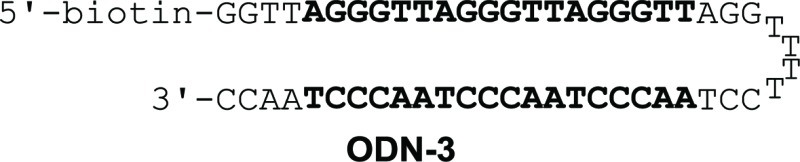	2.0 × 10^5^	3.3 × 10^–4^	1.7 × 10^–9^	0.69	—	9.8 × 10^4^	3.5 × 10^–4^	3.6 × 10^–9^	0.53	—
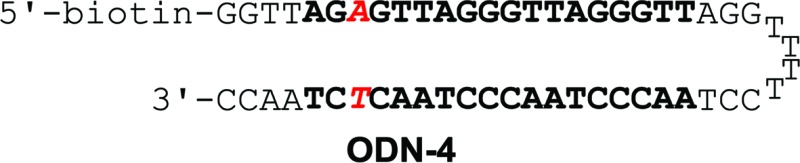	3.4 × 10^5^	6.5 × 10^–4^	1.9 × 10^–9^	0.36	1.1	5.3 × 10^4^	4.9 × 10^–4^	9.3 × 10^–9^	0.60	2.6
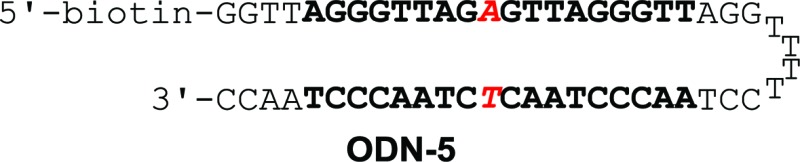	1.3 × 10^5^	3.7 × 10^–4^	2.8 × 10^–9^	0.20	1.6	4.7 × 10^4^	4.2 × 10^–4^	9.0 × 10^–9^	0.33	2.5

^*a*^Specificity was calculated by *K*_D_(mismatch)/*K*_D_(match).

### Human telomere staining with two Py–Im polyamide probes

**Fig. 2:**
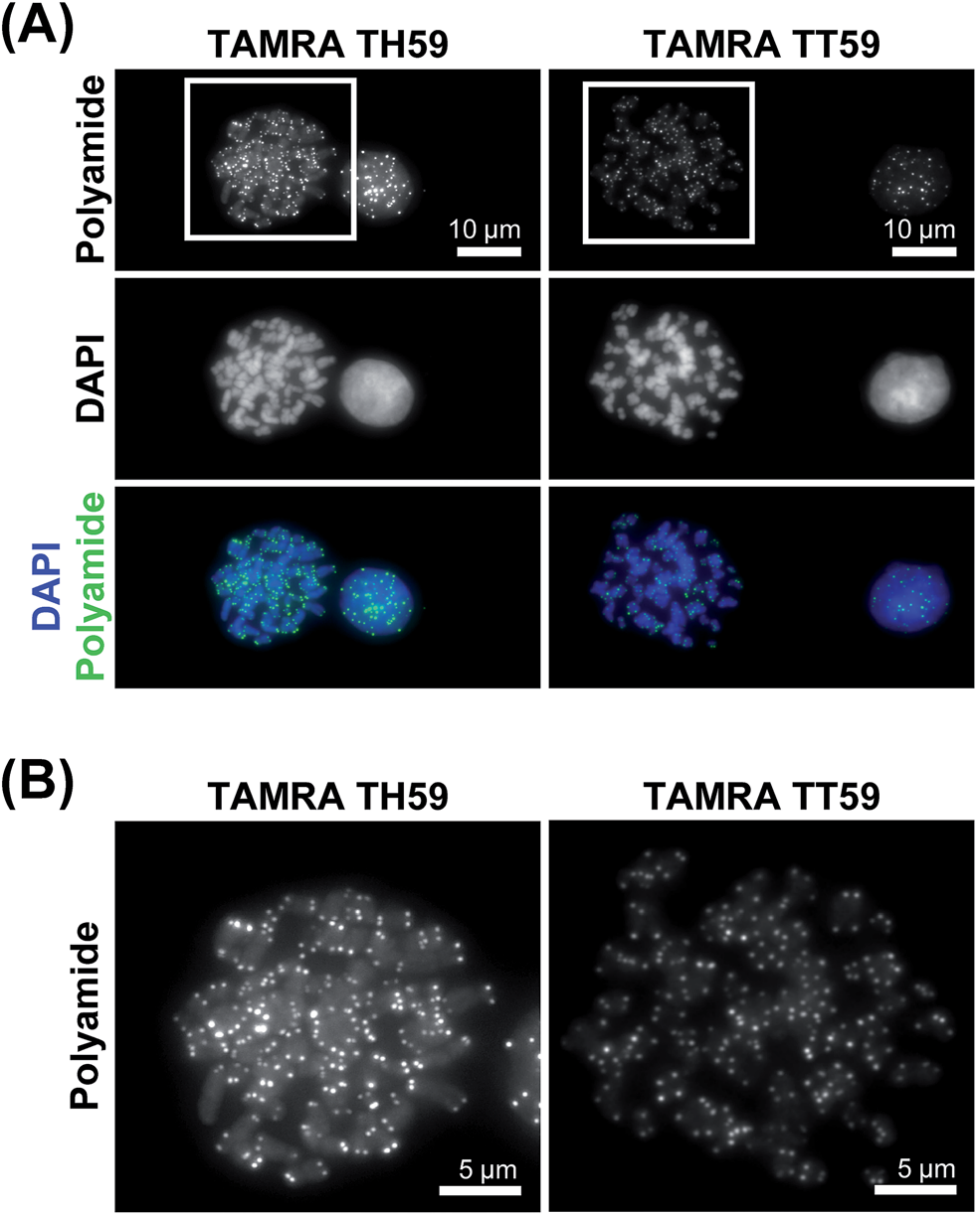
Telomere staining of HeLa 1.3 cell spreads with fluorescent polyamides. (A) The HeLa 1.3 cell spread was stained with the fluorescent polyamide (first row) and DAPI (second row). The merged images are shown in the third row. The first and second columns show the results for **TAMRA TH59** and **TAMRA TT59**, respectively. Enlarged images of the boxed regions in panel (A) are shown in panel (B).

We doubly stained human HeLa 1.3 cell spreads, which are used for clinical karyotype tests, with 4′,6-diamidino-2-phenylindole (DAPI) and Py–Im polyamide probes **TAMRA TH59** and **TAMRA TT59** to compare their abilities to stain telomeres selectively. Cell spreads were prepared by methanol/acetic acid fixation. To ensure a proper comparison between **TAMRA TH59** and **TAMRA TT59**, the images without deconvolution, differently from our previous study,^17*a*^ were presented in Fig. 2. Furthermore, to demonstrate reproducibility of the result, additional image data was depicted in Fig. S6.† DAPI visualized chromosomal regions and nuclei, and Py–Im polyamide probes showed intense foci. Two foci were observed at every chromosomal end, indicating that both probes could stain telomeres selectively. However, the signal intensities appeared to be greater for the probe **TAMRA TH59** (ref. 17*b*) than for **TAMRA TT59**, presumably because more **TAMRA TH59** molecules bound to telomeres compared with **TAMRA TT59**. On the other hand, as shown in Fig. 2B, substantial polyamide signals were observed on the chromosome body when treating cells with **TAMRA TH59**. By contrast, lower-intensity signals on the chromosome body region were observed in the case of **TAMRA TT59**. Background polyamide signals except intense foci derived from nonspecific binding. Cell spreads images indicated **TAMRA TT59** had higher selectivity to telomeres, suggesting that nonspecific binding was reduced by increasing the number of base pairs bound by Py–Im polyamides.

**Fig. 3:**
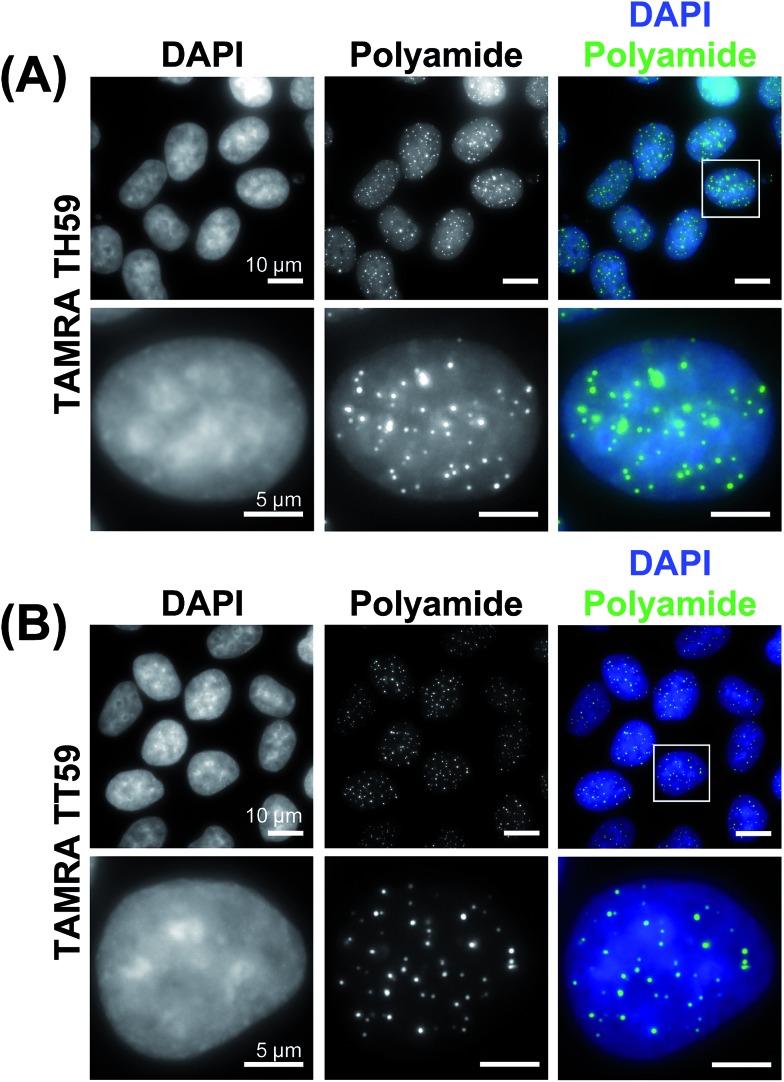
Telomere staining of HeLa 1.3 cells with fluorescent polyamides. HeLa 1.3 cells were stained with DAPI (first column) and fluorescent polyamides (second column). The merged images are shown in the third column. Enlarged images of the boxed regions in the first row are shown in the second row. Cell images using **TAMRA TH59** and **TAMRA TT59** are shown in panels (A) and (B), respectively.

**Fig. 4:**
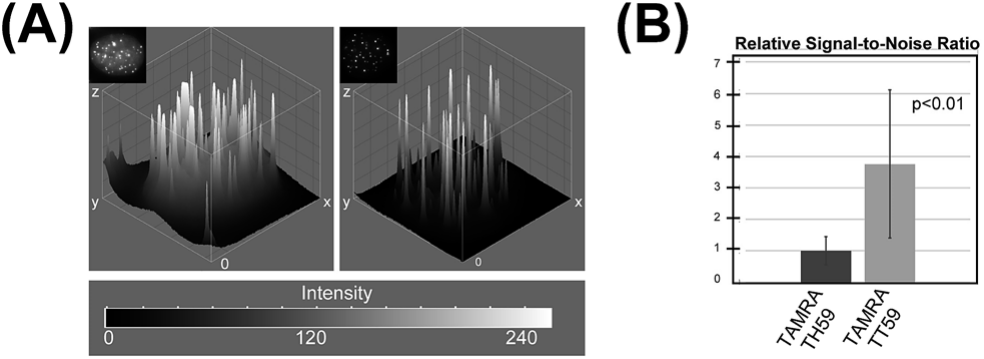
(A) Surface plots based on the boxed images shown in Fig. 3. (B) Relative signal-to-noise ratios of the images.

This result was supported by telomere staining images of HeLa 1.3 cells fixed in formaldehyde (Fig. 3 and S7†), a commonly used fixation method in cell biology. Many sharp polyamide signals were detected by staining with both **TAMRA TH59** and **TAMRA TT59** in nuclei. By contrast, throughout the nucleus, **TAMRA TT59** signals were less intense and rarely merged with DAPI signals. For quantification of the telomere signals, they were extracted from Fig. 3 non-deconvolved images and the results are shown in Fig. 4 as surface plots and signal-to-noise ratio. The sharper surface plots and about 3.7-fold signal-to-noise ratio compared to **TAMRA TH59** also showed lower-intensity background signals of **TAMRA TT59**, suggesting its higher selectivity to telomeres. These images of cell staining were supported by the results of SPR analyses.

## Conclusion

In this study, we have shown the new motif of Py–Im polyamide, which we call a tandem trimer, which comprises three hairpins and two hinge segments and targets 18 bp, the longest reported sequence for Py–Im polyamides. SPR analysis indicated that the tandem trimer Py–Im polyamide bound to 18 bp in human telomere repeats more specifically than did the previous tandem hairpin Py–Im polyamides. Moreover, the TAMRA-conjugated tandem trimer Py–Im polyamide probe highlighted the telomere foci clearly with lower intensity of background signals, showing its higher selectivity to telomeres. This new motif may be useful for highlighting specific regions clearly in human cells.
