# Supplementary material for: Tandem trimer pyrrole–imidazole polyamide probes targeting 18 base pairs in human telomere sequences
Source: Chem Sci. 2015 Jan 20;6(4):2307–12. doi: 10.1039/c4sc03755c (PMC5645774; doi:10.1039/c4sc03755c)
Supplement: Supplementary file 1 [file SC-006-C4SC03755C-s001.pdf]

Electronic Supplementary Material (ESI) for Chemical Science.
This journal is © The Royal Society of Chemistry 2015

Supporting Information

Tandem Trimer Pyrrole-Imidazole Polyamide Probes Targeting 18 Base Pairs in Human Telomere Sequences

Yusuke Kawamoto[†], Asuka Sasaki[‡], Kaori Hashiya[†], Satoru Ide[‡], Toshikazu Bando[*,†], Kazuhiro Maeshima[*,‡], and Hiroshi Sugiyama[*,†,§]

[†]Department of Chemistry, Graduate School of Science, Kyoto University, Sakyo, Kyoto 606-8502, Japan

[‡]Biological Macromolecules Laboratory, Structural Biology Center, National Institute of Genetics, and Department of Genetics, School of Life Science, Graduate University for Advanced Studies (Sokendai), Mishima, Shizuoka 411-8540, Japan.

[§]Institute for Integrated Cell-Material Science (WPI-iCeMS), Kyoto University, Sakyo, Kyoto 606-8501, Japan.

Corresponding authors

(H. S.) hs@kuchem.kyoto-u.ac.jp

(K. M.) kmaeshim@nig.ac.jp

(T. B.) bando@kuchem.kyoto-u.ac.jp

Table of Contents

1. Materials and Methods...S3

2. Sequences of ODN-1 to 5..S13

3. Mass spectra and analytical HPLC profiles of the compounds TH59, TT59, TAMRA TH59 and TAMRA TT59...S14

4. The UV–visible absorption and fluorescence spectra of probes TAMRA TH59 and TAMRA TT59...S16

5. SPR sensorgrams of TH59 and TT59..S17

6. Additional images of telomeres...S19

7. References...S21

1. Materials and Methods

Materials. HPLC analysis was performed on a PU-2089 plus series system (JASCO) using COSMOSIL 4.6 x 150 mm $5C_{18}$-MS-II Packed Column (Nacalai Tesque, Inc.) in 0.1% TFA in water with acetonitrile as the eluent at a flow rate of 1.0 mL/min and a linear gradient elution of 0 to 50% acetonitrile in 20 min with detection at 254 nm. Purifications of Py–Im polyamides are mentioned in each synthetic section. Collected fractions were analyzed by ESI-TOF-MS (Bruker). For the collect mass measurement, external mass calibration (m/z: 622.0290 and 922.0098), purchased from Agilent Technologies, was used. Fmoc-mini-PEG-OH was purchased from Peptide International. Fmoc-Py-CO_2H, *N*,*N*-dimethylformamide (DMF) used for solid-phase synthesis, 1-methyl-2-pyrrolidone (NMP), trifluoroacetic acid (TFA), and piperidine were from Wako. HCTU was from Peptide Institute, Inc. Fmoc-β-Ala-Wang resin (0.60 mmol/g, 100-200 mesh) was from Novabiochem. *N*,*N*-diisopropylethylamine (DIEA) was from Nacalai Tesque, Inc. DMF used for solution-phase synthesis and for telomere-staining and acetonitrile were from Kanto Chemical Co., Inc. 3, 3'-diamino-*N*-methyldipropylamine was from Tokyo Chemical Industry Co., LTD. 5-carboxytetramethylrhodamine succinimidyl ester was from Molecular Probes. The Fmoc units **1**[1] and Fmoc-PyIm-CO_2H[2] were synthesized using the previous method. SPPS was performed on a PSSM-8 (Shimadzu). Dehumidifiers were

from Panasonic. Concentrations of Py–Im Polyamides were calculated with a Nanodrop ND-1000 spectrophotometer (Thermo Fisher Scientific Inc.) using an extinction coefficient of 9900 $M^{-1}cm^{-1}$ per one pyrrole or imidazole moiety at λ_{max} near 310 nm. Measurement of absorption spectra was performed on a Spectophotometer V-650 (JASCO) equipped with a thermocontrolled PAC-743R cell changer (JASCO) and a refrigerated and heating circulator F25-ED (Julabo). Fluorescence spectra were measured on a Spectrofluorometer FP-6300 (JASCO). The SPR assays were performed using a BIACORE X instrument (GE Healthcare) with 5'-biotinylated DNA oligonucleotides (JBioS), streptavidin-coated sensor chip SA (GE Healthcare) and HBS-EP buffer (GE Healthcare). All unmodified DNA oligonucleotides used in this study were purchased from Sigma. Cell images were recorded with DeltaVision (Applied Precision). HeLa 1.3 cells were generous gifts of Dr T. de Lange (Rockefeller University). DMEM medium was purchased from Invitrogen. Normal goat serum (NGS) was from Millipore. Nocodazole, paraphenylene diamine, and Triton X-100 were from Sigma. $NaBH_4$ was from Fluka. DAPI was from Roche. All other solvents and materials were from standard suppliers (highest quality available).

General Procedures of Fmoc Solid-phase Peptide Synthesis. Each synthesis of Py–Im polyamides was performed with a computer-assisted operation system placed in a vinyl

box with dehumidifiers by using Fmoc chemistry under low humidity (< 20%). In the absence of dehumidifiers, the humidity was higher than 40% and the yield of **TH59** was only 6.9%.[1] Furthermore the synthesis of **TT59** was impossible. An Fmoc unit in each step was set up to solve by NMP (1 mL) on the synthetic line. Fmoc units in each step were as follows: Fmoc-Py-CO_2H (77 mg, 0.21 mmol), Fmoc-PyIm-CO_2H (70 mg, 0.14 mmol), Fmoc-D-Dab(ImImPy)-OH (**1**) (70 mg, 0.10 mmol), and Fmoc-mini-PEG-OH (81 mg, 0.21 mmol). Before SPPS, Fmoc units were dissolved in NMP (1.0 mL) with HCTU and the amount of HCTU for Fmoc-Py-CO_2H, Fmoc-PyIm-CO_2H, **1**, and Fmoc-mini-PEG-OH was 88 mg (0.21 mmol), 60 mg (0.15 mmol), 40 mg (0.097 mmol), and 88 mg (0.21 mmol), respectively.

Procedures were as follows: twice deblocking for 4 min with 20 % piperidine/ NMP (0.50 mL), addition of 10% DIEA/NMP (400 μL, DIEA was 0.23 mmol) to Fmoc unit-HCTU mixture for activation, coupling for 60 min. Five times washing with DMF were performed during each step. After the last coupling, the deblocking of amino group at γ-turn was performed. All coupling were carried out with a single-coupling cycle. All lines were purged with solution transfers and bubbled by nitrogen gas for stirring the resin. After solid-phase synthesis, the resin was washed with methanol and dried *in vacuo*, followed by the cleavage of the compound from Wang resin using 0.40 mL 3,3'-diamino-*N*-methyldipropylamine (96 % assay) for 3 h at 55 °C. The resin was removed by filtration

and washed thoroughly with dichloromethane, and the filtrate was concentrated *in vacuo*. The residue was dissolved in 0.50 mL dichloromethane and then 5.0 mL diethyl ether was added to the solution, followed by sonication for a few minutes to obtain the yellow residue. After discarding supernatant, the residue was dissolved in 0.50-1.0 mL dichloromethane-methanol mixture and then more than 10-fold volume of diethyl ether was added, followed by the centrifuge and removal of the supernatant. This process was repeated a few times until white precipitation was obtained. The dried crude precipitation was purified by reversed-phase chromatography.

Synthesis of Tandem Hairpin Py–Im Polyamide TH59. Solid-phase synthesis was performed with 43.0 mg Fmoc-β-Wang resin (0.60 mmol/ g). The resulting crude polyamide (33.7 mg) was purified by reversed-phase flash chromatography. Purification was performed on CombiFlash Rf using a 4.3 g C18 RediSep Rf reversed-phase flash column (Teledyne Isco, Inc.) in 0.1% TFA in water with acetonitrile as the eluent at a flow rate of 18.0 mL/min and a linear gradient elution of 0 to 35% acetonitrile in 5 to 30 min. Collected appropriate fractions were concentrated using an evaporator and then lyophilized to give the polyamide **TH59** (16.9 mg, 7.5 x 10^{-3} mmol, 29 % yield in 10 steps). Analytical HPLC: t_R = 15.2 min. ESI-TOF-MS *m/z* calcd for $C_{102}H_{127}N_{41}O_{20}$ $[M + 2H]^{2+}$ 1124.0169 found 1124.0084 and $[M + 3H]^{3+}$ 749.6805 found 749.6784.

Synthesis of Tandem Trimer Py–Im Polyamide TT59. Solid-phase synthesis was performed with 47.0 mg Fmoc-β-Wang resin (0.60 mmol/ g). The resulting crude polyamide (33.6 mg) was purified by PU-2089 series HPLC (JASCO) using COSMOSIL 10 x 150 mm 5C_{18}-MS-II Packed Column (Nacalai Tesque, Inc.) in 0.1% TFA in water with acetonitrile as the eluent at a flow rate of 3.0 mL/min and a linear gradient elution of 25 to 32.5% acetonitrile in 20 min. The collected fractions were treated as described above to give the polyamide **TT59** (8.0 mg, 2.4 x 10^{-3} mmol, 8.5% yield in 15 steps). Analytical HPLC: t_R = 16.0 min. ESI-TOF-MS *m/z* calcd for $C_{151}H_{184}N_{60}O_{31}$ $[M + 3H]^{3+}$ 1112.1634 found 1112.1313, $[M + 4H]^{4+}$ 834.3745 found 834.3653 and $[M + 5H]^{5+}$ 667.7011 found 667.7015.

Synthesis of the Florescent Probe TAMRA TH59. The polyamide **TH59** (1.3 mg, 5.8 x 10^{-4} mmol) and 5-carboxytetramethylrhodamine succinimidyl ester (0.60 mg, 1.1 x 10^{-3} mmol) were dissolved in DMF (300 μL) and DIEA (0.40 μL, 2.3 x 10^{-3} mmol), followed by mixing for 4 h at room temperature with shielding the light. The reaction mixture was purified by reversed-phase HPLC performed on a UV2075 HPLC UV/VIS detector and a PU-2080 plus series system (JASCO) using CHEMCOBOND 4.6 mm x 150 mm 5-ODS-H Column (Chemco) in 0.1% TFA in water with acetonitrile as the eluent at a flow rate of

1.0 mL/min and a linear gradient elution of 30 to 50% acetonitrile in 15 min. Appropriate fractions were collected under freeze-dry conditions to give the probe **TAMRA TH59** (1.6 mg) as a purple powder. Analytical HPLC: t_R = 16.4 min. ESI-TOF-MS *m/z* calcd for $C_{127}H_{147}N_{43}O_{24}$ $[M + 2H]^{2+}$ 1330.0881 found 1330.0630, $[M + 3H]^{3+}$ 887.0613 found 887.0511 and $[M + 4H]^{4+}$ 665.5479 found 665.5453.

Synthesis of the Florescent Probe TAMRA TT59. The polyamide **TT59** (0.93 mg, 2.8×10^{-4} mmol) and 5-carboxytetramethylrhodamine succinimidyl ester (0.35 mg, 6.6×10^{-4} mmol) were dissolved in DMF (200 μL) and DIEA (0.20 μL, 1.1×10^{-3} mmol), followed by mixing for 4 h at room temperature with shielding the light. The reaction mixture was purified as described above to give the probe **TAMRA TT59** (1.7 mg) as a purple powder. Analytical HPLC: t_R = 17.0 min. ESI-TOF-MS *m/z* calcd for $C_{176}H_{204}N_{62}O_{35}$ $[M + 3H]^{3+}$ 1249.5441 found 1249.5127 and $[M + 4H]^{4+}$ 937.4101 found 937.3922.

Absorption and Fluorescence Spectra. All spectra of fluorescent probes **TAMRA TH59** and **TAMRA TT59** were obtained in 7 mM sodium phosphate buffer (pH 7.0) containing 5.0% v/v DMF at room temperature. The UV–visible absorption spectra of 6.0 μM probes were obtained and steady state fluorescence spectra of 3.0 μM probes were

measured. Steady state fluorescence spectra of 3.0 μM probes were obtained with an excitation wavelength of 540 nm in the absence or presence of 1.0 eq. or 2.0 eq. of ODN-1/2.

SPR Assays. The SPR assays were performed as described in previous reports.[3,4] Biotinylated DNA was immobilized to streptavidin-coated sensor chip SA at a flow rate of 10 μL/min to obtain the desired immobilization level (up to approximately 1400 RU rise). The assays were performed with HBS-EP (10 mM HEPES pH 7.4, 150 mM NaCl, 3 mM EDTA, 0.005% v/v Surfactant P20) with 0.1% DMSO at 25 °C. A series of sample solutions with various concentrations were prepared in HBS-EP buffer with 0.1% DMSO and injected at a flow rate of 20 μL/min. To calculate association rates (k_a), dissociation rates (k_d), and dissociation constants (K_D), data processing was performed with 1:1 binding with mass transfer model using BIAevaluation 4.1 program. The closeness of the fitting lines is described as χ^2 shown in the following equation. r_f is the fitted value at a given point, r_x is the experimental value at the same point, n is the number of data points, and p is the number of fitted parameters.

$$\chi^2 = \frac{\sum_1^n (r_f - r_x)^2}{n - p}$$

Telomeres Staining

Telomere Staining of HeLa 1.3 Cell Spreads with Fluorescent Polyamides.[3,5] HeLa 1.3 cells were maintained in DMEM medium containing 10% fetal bovine serum (FBS) at 37 °C (5% CO_2). HeLa 1.3 cells were blocked mitotically by adding 0.1 μg/mL nocodazole for 3 h. The cells were swollen by treatment with a hypotonic buffer (0.075 M KCl) for 15 min at room temperature and then fixed with a methanol/acetic acid (3:1) solution for 5 min. After centrifugation, cell pellet was again suspended in the methanol/acetic acid solution. The cell suspension was spread on coverslips and air-dried for 30 min. The cell spread coverslips were kept at 4 °C until use.

The air-dried spread coverslips were soaked in TEN buffer (10 mM Tris-HCl, pH 7.5, 1 mM EDTA and 100 mM NaCl) over night at 4 °C before use. For blocking, the spread coverslips were treated with 10% normal goat serum (NGS) in TEN buffer for 30 min at room temperature. After brief washing with TE buffer (10 mM Tris-HCl, pH 7.5, 1 mM EDTA), the spread coverslips were incubated with 10% NGS, 10 nM **TAMRA TH59** or 50 nM **TAMRA TT59** in DMF, and 0.5 μg/ml DAPI in TE buffer for 1 h at room temperature. After washing with TEN buffer (five times for 3 min), the spread coverslips were mounted in PPDI solution [10 mM HEPES pH 7.5, 100 mM KCl, 1 mM $MgCl_2$, 80% glycerol, 1 mg/mL paraphenylene diamine][5] and the coverslips were sealed with a nailporish. Sectioning images were recorded with DeltaVision and projected ("Quick

Projection" tool) without deconvolution to obtain telomeric signals and background signals.

Telomere Staining of Formaldehyde-fixed HeLa 1.3 Cells with Fluorescent Polyamides.[3] For polyamide staining, the HeLa cells were grown on coverslips coated with polylysine. The cell coverslips were washed in phosphate-buffered saline (PBS) twice and fixed with 2% formaldehyde in PBS for 15 min at room temperature. The fixed cell coverslips were then treated with 50 mM glycine in PBS for 5 min, rinsed with PBS (twice for 5 min) and permeabilized with 0.5% Triton X-100 in PBS for 5 min. After briefly washing with HMK buffer (10 mM HEPES pH 7.5, 1 mM $MgCl_2$, 100 mM KCl) twice, the coverslips were soaked in HEN buffer (10 mM HEPES pH 7.5, 1 mM EDTA, 100 mM NaCl) and kept at 4°C until use. For blocking, the cell coverslips were treated with 10% NGS in TE buffer (10 mM Tris-HCl pH 7.5, 1 mM EDTA) for 30 min at room temperature. After a brief rinse with TE buffer, the cells were incubated with 10% NGS, 10 nM **TAMRA TH59** or 50 nM **TAMRA TT59** in DMF, and 0.5 μg/mL DAPI in TE buffer at 37 °C for 1 h. After washing with TEN200 buffer (10 mM Tris-HCl pH 7.5, 1 mM EDTA, and 200 mM NaCl) (five times for 3 min). The mounting and subsequent image acquisitions were performed as described above. For quantification of the telomere signals in Figure 4, they were extracted from the Figure 3 images as described previously.[6]

The telomere signals yielded by the polyamides were extracted based on threshold values using the Softworks software (Applied Precision). The maximum intensity values of signals in the extracted telomere regions were then used as telomere signals. For the background signals, 10 squares (10 pixels x 10 pixels) were randomly set outside the defined telomere signals and mean values of the signals in the squares were used as background signals.

2. Sequences of ODN-1 to -5

**ODN-1**	5′-GGTTAGGGTTAGGGTTAGGGTTAGG-3′
**ODN-2**	5′-CCTAACCCTAACCCTAACCCTAACC-3′
**ODN-3**	5'-biotin-GGTTAGGGTTAGGGTTAGGGTTAGGTTTTCCTAACCCTAACCCTAACCCTAACC-3'
**ODN-4**	5'-biotin-GGTTAG***A***GTTAGGGTTAGGGTTAGGTTTTCCTAACCCTAACCCTAAC***T***CTAACC-3'
**ODN-5**	5'-biotin-GGTTAGGGTTAG***A***GTTAGGGTTAGGTTTTCCTAACCCTAAC***T***CTAACCCTAACC-3'

Table S1. Sequence of the oligonucleotides. Underlined bases are binding sites of **TT59** and bold, italicized bases are mismatch sites for **TT59**.

3. Mass spectra and analytical HPLC profiles of the compounds TH59, TT59, TAMRA TH59 and TAMRA TT59

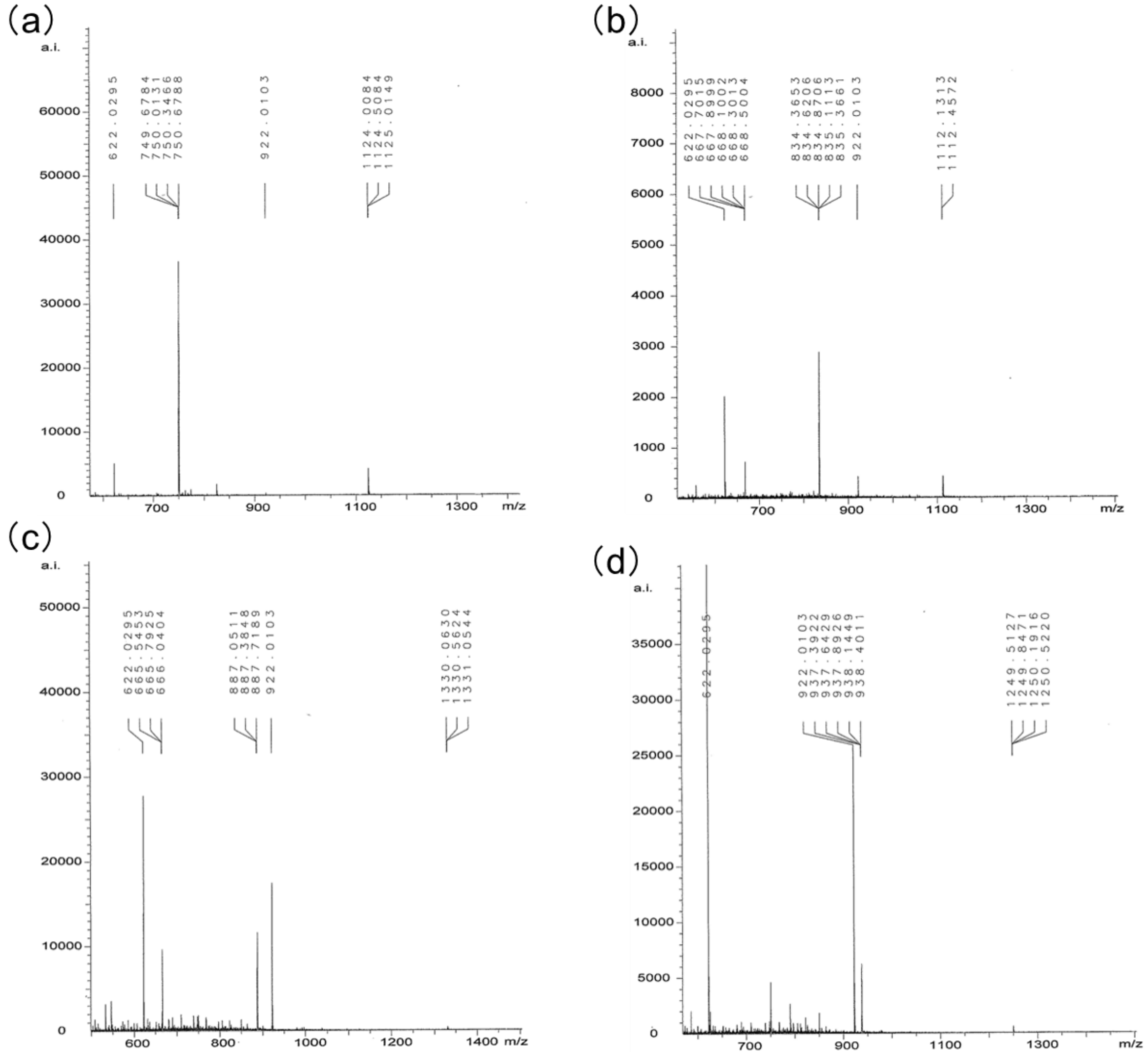

Figure S1. ESI-TOF MS spectra of the polyamides **TH59**, **TT59**, **TAMRA TH59** and **TAMRA TT59** (a: **TH59**, b: **TT59**, c: **TAMRA TH59**, d: **TAMRA TT59**).

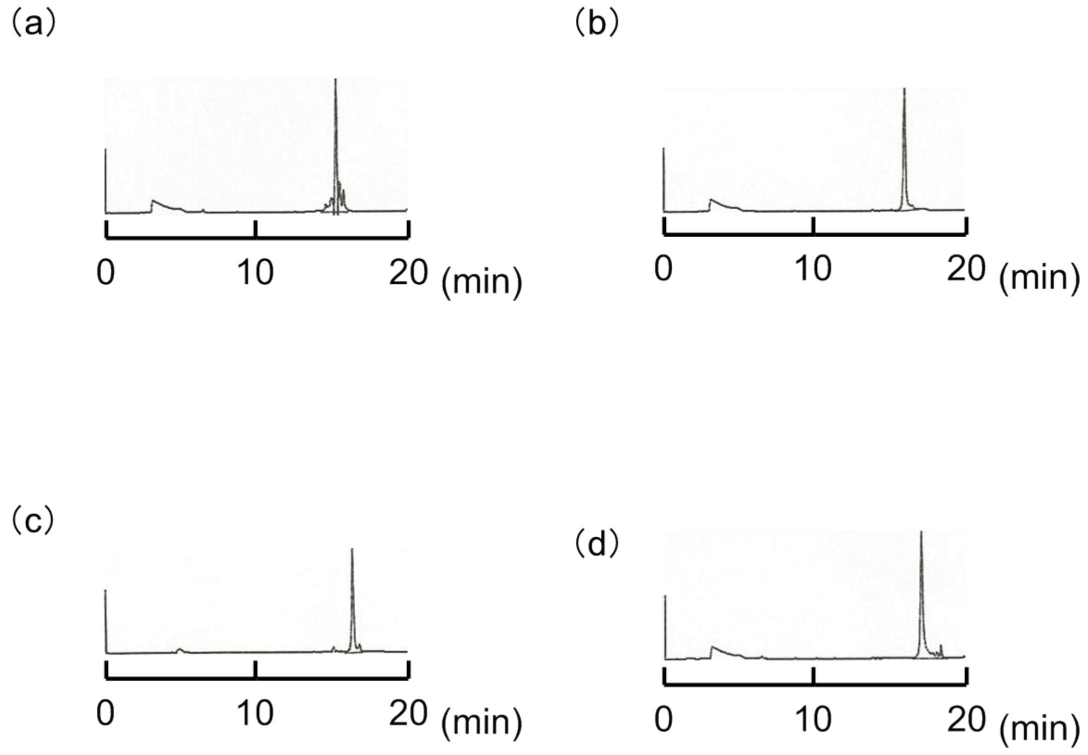

Figure S2. Analytical HPLC profiles of the polyamides **TH59**, **TT59**, **TAMRA TH59** and **TAMRA TT59** (a: **TH59**, b: **TT59**, c: **TAMRA TH59**, d: **TAMRA TT59**).

4. The UV–visible absorption and fluorescence spectra of probes TAMRA TH59 and TAMRA TT59

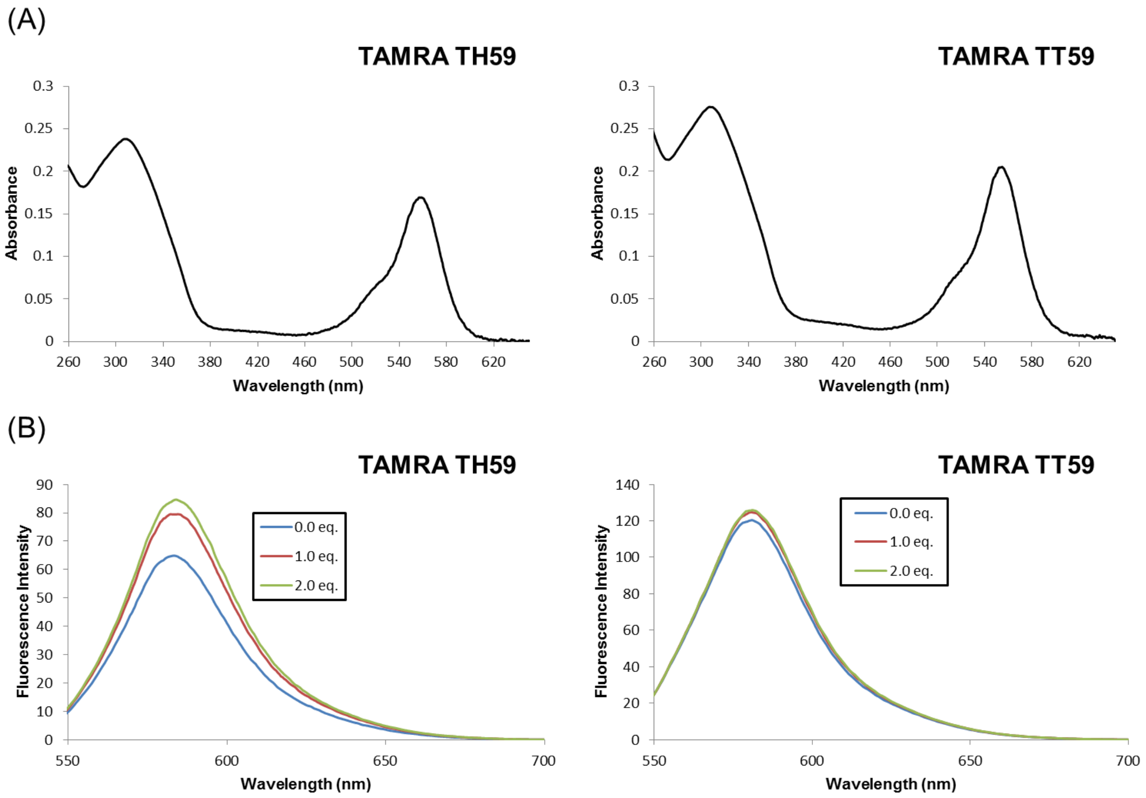

Figure S3. (A) UV–visible absorption spectra of 6.0 μM probes **TAMRA TH59** and **TAMRA TT59** and (B) fluorescence spectra (λ_{ex} = 540 nm) of 3.0 μM probes **TAMRA TH59** and **TAMRA TT59** in the absence (blue) or presence of 1.0 equivalent (red) or 2.0 equivalent ODN-1/2 (green). Each spectrum was measured in 7 mM sodium phosphate buffer (pH 7.0) containing 5% v/v DMF.

5. SPR sensorgrams of TH59 and TT59

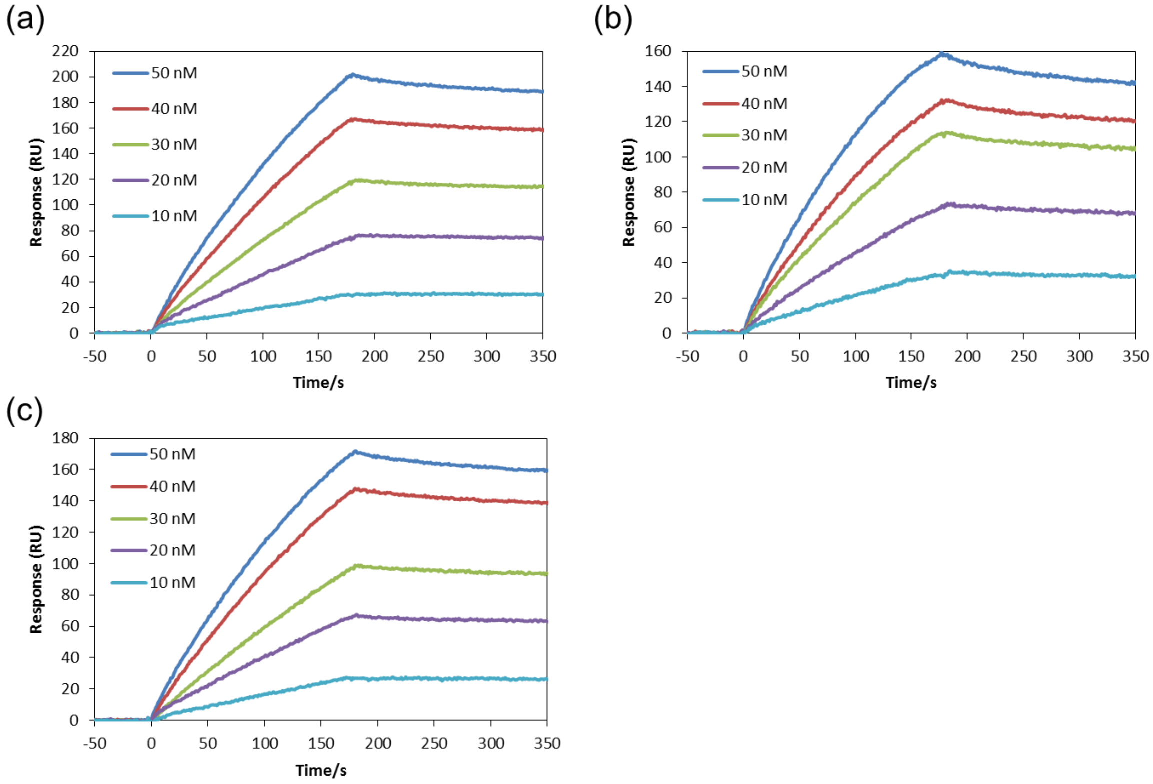

Figure S4. SPR sensorgrams for the interaction of the polyamide **TH59** and (a) ODN-3, (b) ODN-4 or (c) ODN-5.

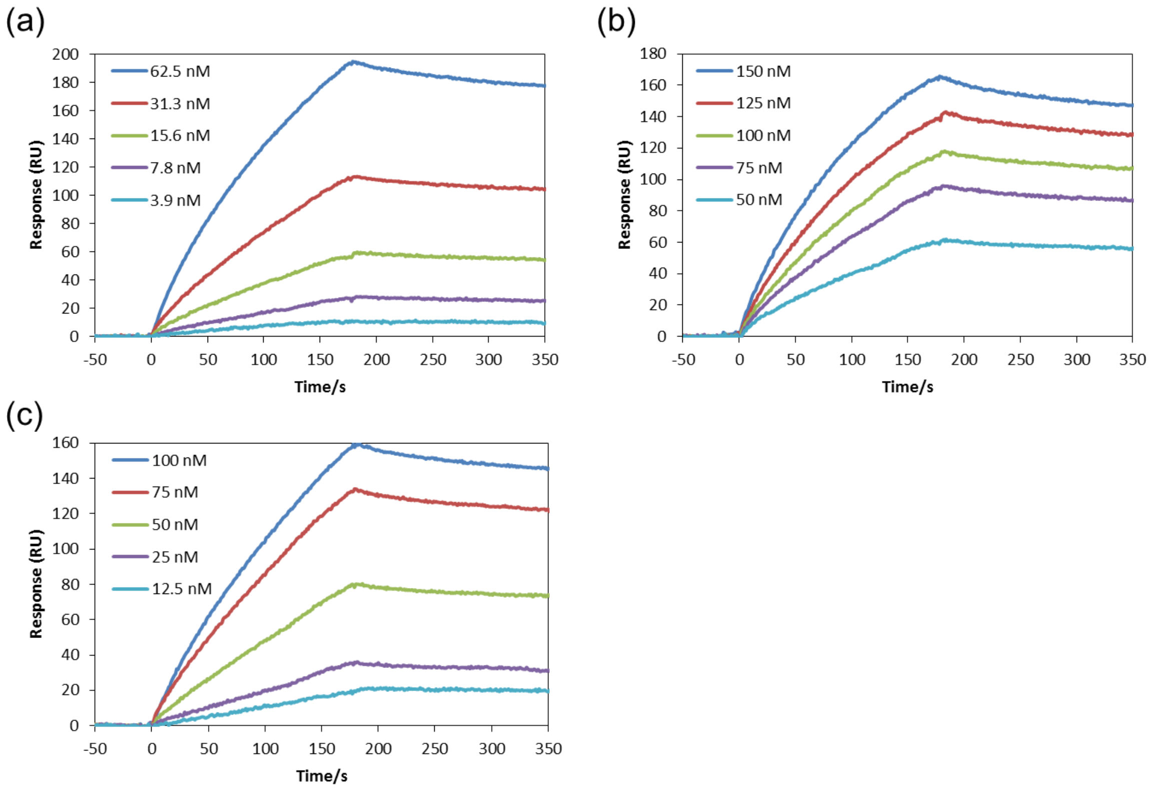

Figure S5. SPR sensorgrams for the interaction of the polyamide **TT59** and (a) ODN-3, (b) ODN-4 or (c) ODN-5.

6. Additional images of telomeres

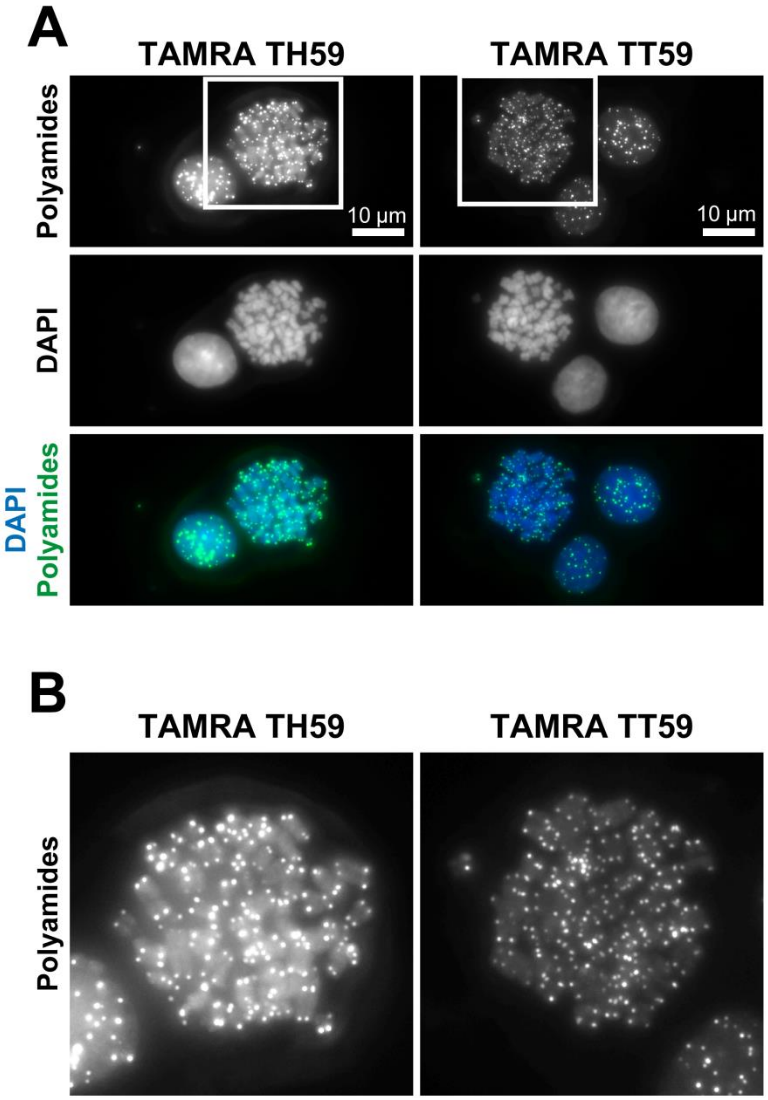

Figure S6. Telomere staining of HeLa 1.3 cell spreads with fluorescent polyamides. (A) The HeLa 1.3 cell spread was stained with the fluorescent polyamide (first row) and DAPI (second row). The merged images are shown in the third row. The first and second columns show the results for **TAMRA TH59** and **TAMRA TT59**, respectively. Enlarged images of the boxed regions in panel A are shown in panel B.

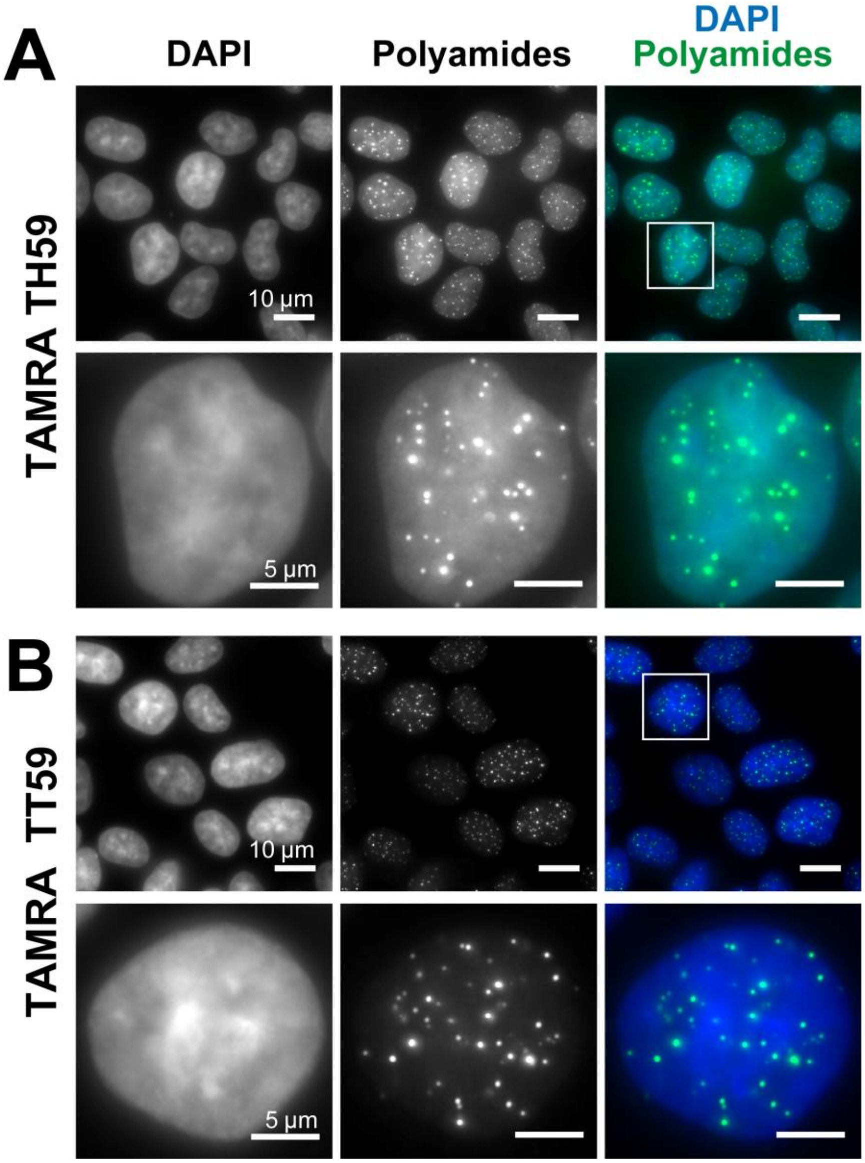

Figure S7. Telomere staining of formaldehyde-fixed HeLa 1.3 cells with fluorescent polyamides. HeLa 1.3 cells were stained with DAPI (first column) and fluorescent polyamides (second column). The merged images are shown in the third column. Enlarged images of the boxed regions in the first row are shown in the second row. Cell images using **TAMRA TH59** and **TAMRA TT59** are shown in panels A and B, respectively.

7. References

1. Y. Kawamoto, T. Bando, F. Kamada, Y. Li, K. Hashiya, K. Maeshima, H. Sugiyama, *J. Am. Chem. Soc.*, 2013, **135**, 16468-16477.

2. M. Minoshima, T. Bando, S. Sasaki, J. Fujimoto, H. Sugiyama, *Nucleic Acids Res.*, 2008, **36**, 2889-2894.

3. A. Hirata, K. Nokihara, Y. Kawamoto, T. Bando, A. Sasaki, S. Ide, K. Maeshima, T. Kasama, H. Sugiyama, *J. Am. Chem. Soc.*, 2014, **136**, 11546-11554.

4. E. R. Lacy, N. M. Le, C. A. Price, M. Lee, W. D. Wilson, *J. Am. Chem. Soc.*, 2002, **124**, 2153–2163.

5. (a) K. Maeshima, U. K. Laemmli, *Dev. Cell*, 2003, **4**, 467-80. (b) K. Maeshima, K. Yahata, Y. Sasaki, R. Nakatomi, T. Tachibana, T. Hashikawa, F. Imamoto, N. Imamoto, *J. Cell Sci.*, 2006, **119**, 4442-4451.

6. K. Maeshima, S. Janssen, U. K. Laemmli, *EMBO J.*, 2001, **20**, 3218-3228.
